# Competent Witnesses: How Penitentiary Workers Explain the Violence in Italian Prisons during the COVID-19 Pandemic

**DOI:** 10.3390/ijerph192113717

**Published:** 2022-10-22

**Authors:** Ines Testoni, Davide Viezzoli, Gianmarco Biancalani, Maria Armezzani, Adriano Zamperini

**Affiliations:** FISPPA Department, University of Padova, 35131 Padova, Italy

**Keywords:** prison personnel, prison violence, detainees, psychosocial and relational competence, COVID-19

## Abstract

Background: During the COVID-19 pandemic, in the Italian prison of Santa Maria Capua Vetere (SMCV), prison police repressed a riot with extreme violence, bringing the state of prisons and the conditions of prisoners back to the attention of the Italian public opinion. Objective: This exploratory study aimed to collect the experiences and the competent opinions of the social and health personnel of Italian prisons regarding the episode of violence that happened in SMCV; the general state of health of the Italian prison system was explored, too, together with the collection of proposals for interventions aimed at the eradication of violence in prison. Method: The study employed a qualitative research design. Eighteen social-health workers from 12 Italian prisons were interviewed using in-depth interviews of ~60 min each that were conducted and recorded via Skype video calls. The interview transcripts were analyzed with qualitative reflexive thematic analysis (RTA) to identify the most relevant and recursive themes. Results: Four themes were identified: (1) reactions and thoughts about the events of SMCV; (2) structural problems of Italian prison police; (3) Italian prison system; and (4) reform proposals. Conclusions: A new and deeper awareness of the suffering of the current Italian penitentiary system emerged, together with courageous reform proposals that can restore dignity and centrality to the re-education of the detainees, preventing further future violence.

## 1. Introduction

The Italian government is perennially engaged in the effort to change the dramatic penitentiary situation, in agreement with the European Union instances. Indeed, because of dehumanizing conditions and treatments, overcrowding, and the high level of suicide of the detained population, this country was already condemned by the second section of the European Court for violating Article 3 of the European Convention on Human Rights [1]. Since this sentence, the efforts of successive governments have tried and are still trying to improve the situation, for example, by decreasing the crowding inside the cells and implementing the possibilities of probation, which allow those convicted of crimes to avoid serving time in prison. The goal of these solutions is, on the one hand, to make the prison environment less dehumanizing and, on the other hand, reducing the likelihood that prisoners will recommit new crimes [2]. One of the most limiting aspects of these types of changes is that the population of those interacting within the penitentiary system is not substantially changed. Those who undergo transformations in the mode of sentence execution alone are the inmates. Prison workers remain the same with the same competencies and this fossilization produces effects. In fact, it seems that the Italian government intends to keep the system of punishment of the offenders unchanged, simply reducing the risk of incurring new international sanctions with minimal changes, not always supported by sufficient social devices capable of ensuring the success of the enterprise. The condition of serious suffering and the growing fragility of the Italian prison population can be highlighted with the data resulting from the number of suicides of prisoners in the first 8 months of 2022, which increased by 47% compared to the previous year [3]. The inadequacy of this type of intervention inevitably comes to light in the most critical situations, as, for example, with the COVID-19 pandemic.

During the early stages of the COVID-19 pandemic, Italy found itself in complete lockdown as a measure to prevent the spread of the virus [4]. Marginal places such as prisons immediately began to show signs of unease, especially moved by fear of contagion, given the general conditions of overcrowding (to date the rate is 113.1%; [5]) and the sudden ban on visits from the inmates’ relatives [6]. Dealing with a virus in an already overcrowded context unveiled the lack of competences, organization, and facilities, and led inmates to higher levels of distress and anxiety related to death [6]. These measures prompted a series of riots that broke out in many prisons: out of a total of 189 prisons, over 70 were affected by serious structural damage resulting from vandalism and arson, while 30 prisons held peaceful demonstrations [7]. The fundamental objective of this paper is to highlight how it is necessary, in the Italian prison system, to systemically modify the entire relational system, intervening not only with the prisoners but also with those who interact with them in the pedagogical area, such as psychologists, educators, and social workers.

To get into the merits of this, we wanted to consider a specific critical situation, that of “Francesco Uccella” prison of Santa Maria Capua Vetere [SMCV]. During the very first phase of the COVID-19 pandemic, on the evening of April 5th, a protest began inside the prison and it ended with an agreement between the prison management and the inmates on the same night. What happened the following day, 6 April 2020, is currently subject to investigation by SMCV’s Prosecution [7]. The footage recorded by the cameras of the prison and transmitted on 29 June 2021 by an online newspaper, showed 283 agents with balaclavas indiscriminately hammering with batons, slaps, and helmeted headbutts all the inmates [8]. For the repression pursued by penitentiary police, defined as “a horrible slaughter” [8] by SMCV’s prosecutor, 105 among the agents, operators, and officials of the Department of Penitentiary Administration are currently accused and will be prosecuted for the felony of torture, injuries, abuse of authority, forgery, and cooperation in manslaughter.

These dire events can be attributed to the abysmal conditions of Italian prisons that stem from overcrowding, which makes for high constant stress levels of inmates and custodial workers. We define a critical event as any situation that can severely challenge professionals who have to face a situation that requires skills they do not have, directly or indirectly, resulting in concern [9]. A critical event is generally an unexpected event, given its low frequency [10], which deprives professionals of the feeling of being in control of the situation and is characterized by a perception of danger for their psychological or physical well-being. This type of event constitutes a threat to the individual’s well-being [10].

These factors are also classified by the Department of Penitentiary Administration at the Department of Justice and include calamities that can compromise the well-being of the prison community, such as the COVID-19 emergency. This premise makes it possible to qualify the impact of a critical event based on the subjective perception of the people involved. Unfortunately, there is no research on this specific issue.

### 1.1. Theoretical Background

The role of the witness in a violent contest is present in a vast amount of the literature. The competence of commentators, who can assume the role of witnesses to an event in order to judge it, has mostly been considered in the juridical field. The tradition in this field of study is very long and the literature is also very specific [11]. The basic problem that is considered in this area of studies is inherent, on the one hand, to the credibility of the witness (i.e., the competency of children or mentally disabled people to understand a criminal event [12,13] or memory distortion processes [13]) and, on the other hand, to the medical or psychological experts commenting on the criminal event [14,15]. Specifically, forensic psychology and psychiatry [16,17], forensic anthropology [18], and forensic medicine [19] have developed a number of areas of expertise to offer specific interpretations of criminal facts in courtrooms. On the other hand, in the area of professional life within institutions, the practice of expert judgment has developed in the health care field along two directions: the study for the prevention of medical errors [20] and the practice of psychological supervision for the prevention of work-related distress [21]. The literature related to competencies to recognize and prevent errors in prison is scarce and mostly related to suicide prevention among inmates and among prison police officers [22]. There is still a lack of the systematic literature that would enable the expertise of psychologists, educators, and social workers working in prisons to be brought to bear on the prevention of violence. Prevention that can be achieved without increasing the levels of restraint of inmates and those who work with them, including prison police.

The literature on police violence against prisoners is relatively scant. In the psychological field, the Stanford Prison Experiment (SPE) and the BBC Study, albeit criticized for a design neither rigorous nor immune to bias [23,24,25] remain a classic in the description of the dynamics of power established in closed and total institutions such as prisons [26,27]. A systematic review [28] on studies concerning general violence in US prisons over the past 50 years suggests a strong imprint of the prison environment in generating violent episodes: the greater the deprivation, the more a culture of violence is rooted in the prison population. Violence apparently takes root more easily in less open, more controlled institutions, with fewer privileges for prisoners and less tolerant staff [29]. In the Italian context, the decision-making practices of operators in a maximum security prison were analyzed [30], noticing a triple form of structural violence against prisoners: the violence of inertia, wherever in the management of the prisoner the component of violence is taken for granted; bureaucratic violence, when administrative intricacy serves as a frustrating instrument of discipline or retaliation; and the ambiguity of protection—for example, when the management of suicide attempts turns into violence that causes even more suffering. Another form of violence that prisoners are exposed to is failure of accountability, when institutional communication indulges in blaming the violence on the “bad apples” of an otherwise healthy system; such a form of violence minimizes the damage suffered by prisoners in a process of nonlegitimation of pain propagating in an endless victimization [31].

On the side of the prison policemen, the studies focus on the cultural and emotional architectures of the group and internal dynamics of the military corps. Agents tend to perceive themselves as poorly recognized professionally, tending to seek redemption with a deployment of strength and power [32]. They also implement mechanisms of confrontation and denial against an external “rival” group—criminals and prisoners—which allows for a better definition of their own—that of justice. Some help in this comes from the uniform, whose symbolic importance allows to separate from the different, to mentally prepare for a task and to provide psychological protection [33]. Environmental stressors of prison combined with rigid mentality and the impossibility to share sufferance for fear of stigma [34] favors the development of mental illness in agents [35,36]. A recent work by Testoni and colleagues [37] on prison police agents found that approximately 30% of the sample met the criteria for burnout syndrome; a high fear of suicide, a construct that is generally linked to suicidal ideation [38], appeared, too. The hypothesis was also confirmed that the tendency to attribute dehumanizing traits to inmates related to the increase in the attribution of humanizing traits to fellow policemen [37].

### 1.2. The Present Study

The general objective of this exploratory study was the systematic and analytical collection of the opinions of the prison operators of several Italian penitentiaries, in the light of their experience inside a prison, about the facts of SMCV: how and why it could have happened. Starting from this first interpretation, more specific objectives were the collection of experiences and competencies about the current state of health of Italian prisons, from an organizational and relational point of view. The choice of participants fell on the operators of the pedagogical area of various prisons as their expert and attentive gaze rests both on the prisoners and on the prison police officers, of whom they are colleagues. Specifically, we wanted to understand whether prison operators were aware of the fragility of the inmates they worked with, especially during the hardest phase of the COVID-19 pandemic. Ultimately, the collection of the resulting proposals for improvements was aimed at eradicating violence in prison and successfully reintegrating former convicted into society.

## 2. Materials and Methods

### 2.1. The Participants

A convenience sample was recruited of 18 female social-health workers with a mean length of service of 18.22 years (range: from 3 to 37 years), as different professional figures, in 12 different penitentiaries from Northern and Central Italy (see Table 1). Participants agreed to take part in this study and were interviewed by one of the authors, who transcribed the interviews verbatim. To ensure the anonymity of each participant, fictitious names were assigned during the transcription of the interviews.

### 2.2. Recruitment

A snowball sampling method was used to recruit participants. Snowball sampling is a nonprobability sampling method consisting of two steps: identifying potential subjects in the population and asking those to either recruit or suggest to the researcher other potential participants. These steps are repeated until the needed sample size is found [39]. In the case of this study, participants were recruited via email or telephone and informed of the objective and methods of the research. Every participant gave the authors other names of professional figures to make contact with. The inclusion criteria assumed having worked in the Italian prison system as a social or health operator for at least 3 years and to be fluent in Italian. The social and health workers of the “Francesco Uccella” institute of SMCV were not contacted since they are persons of interest, and the matter is sub judice.

Participants did not receive any compensation for taking part in the interviews.

All participants provided their informed consent to participate in the study. All the participants’ names have been changed and all the disclosed content has been modified, including the names of the prisons the participants work at. Only the geographical origin of the institutes (North, Central, or Southern Italy) is generically indicated. Participants were informed of the study aims and procedures, were told that participation was voluntary and that they could withdraw from the study at any time. Permission to record, transcribe, and analyze the conversation was also granted. This study adhered to the American Psychological Association’s ethical principles and Code of Conduct, as well as the principles of the Declaration of Helsinki. It was approved by the Ethics Committee of the University of Padua (Prot. n. BB4DCE00A75F9FC621E922D1B98E00AB).

### 2.3. Data Collection

The interviews were conducted by one of the authors via online platform (Google Meet, Skype, or Zoom) during the three months following the events of SMCV. The choice of the channel through which to conduct the interview had been previously agreed with each of the participants. The interviews were conducted according to the interview method of the Interview focused on the topic [Intervista centrata sul tema], namely, an interview focused on the subject of the research (Armezzani, being written). The initial phase of the interview consisted of providing participants with several triads of statements made by public figures regarding the subject of interest, chosen by the interviewer. The three statements could either be openly contrasting each other or differing for tiny shades of meaning. Therefore, the participant was invited to choose the statement she felt most represented by or, on the contrary, the statement that mostly differed from her view. Contrasts or analogies with statements of third parties facilitate the participants to start a free conversation, beyond the stakes traced by pre-established questions, to result in a dialogic configuration. Specifically for the present research, it was decided to present to the participants two triads of statements made by public and recognizable figures. In the preparatory phase, the main statements of such public figures on the facts of SMCV were, therefore, examined. From the first phase of identification, in which 15 statements were collected, 6 were chosen, based on criteria of clarity and representativeness of a specific line of thought. The six selected citations were subsequently divided into two triads and, thus, presented to the participants. The first three allowed the participant to immediately identify the three main interpretations that emerged in the media about the possible reasons that led to the escalation of violence in the Campania prison. The statements provided to participants were the following:

1. “What happened in Santa Maria Capua Vetere was the result of the frustration of the police”;
2. “As long as the individual policeman feels covered by a higher authority when exerting violence, it will not be possible to speak of ‘bad apples’, rather of a systemic problem”;
3. “It was supposed to be a normal search after the riots; the situation just got out of hand”.

The following three opinions summarized three experts’ lines of thought:

1. “The only hope is to dismantle the prison institution, to free the policemen and allow a path of real recovery to the prisoners”;
2. “It is necessary to have video surveillance systems everywhere, in order to protect not only the prisoners’, but also the prison police officers’ security”;
3. “We need new hires, new uniforms, new cameras, more defence and dialogue instruments for the Penitentiary Police”.

Participants were then left free to choose the sentence they were either more in agreement or more in disagreement with. They were, therefore, invited to explain and comment on the causes of their choice, as a free dialogue was started with the interviewer.

### 2.4. Data Analysis

Thematic analysis (TA) [40] was adopted on the transcripts of the individual interviews. TA is a method used to identify, analyze, and report the recurring themes within the research data, organizing and describing the results that emerged in detail [40]. TA is well-suited for studying participants’ narratives in terms of emotions, experiences, and perceptions [40]. The thematic analysis was conducted according to the six phases outlined by Braun and Clarke [40]: familiarization with the data, coding, generating initial themes, reviewing themes, defining and naming themes, and writing up the report. For the purposes of the present study, an inductive approach to TA was needed. Inductive TA is a data-driven process; hence, it is a qualitative design that allows researchers to codify the data without a pre-existing coding frame [40]. In particular, in this design, the reflexive approach to TA was adopted [41]. Reflexive thematic analysis highlights the researcher’s active role, as codes represent the researcher’s interpretation of patterns across the data. Therefore, this approach stands as “the researcher’s reflective and thoughtful engagement with their data and their reflexive and thoughtful engagement with the analytic process” ([41], p. 594). To complete the analysis, Atlas.ti [42] was used. Atlas.ti is a software which allows the researchers to work directly on the texts of the interviews, facilitating the process of qualitative analysis.

## 3. Results

### 3.1. Theme 1: Interpretations and Feelings about the Events of Santa Maria Capua Vetere

Participants expressed their ideas on how and why the situation of the “Francesco Uccella” prison plunged into a violent escalation. Anna, a clinical criminologist who has worked in the penitentiary system for 3 years, underlined the importance of hierarchy in the decision-making system of a penitentiary and that this may have had an influence on the course of events of violence:

“It was necessarily legitimized by higher authorities […]. It is structurally impossible to do this [the mass beating] if a commander, the director or the supervisor himself are aware of and oppose it. More than 280 agents… it can’t be done, it’s too big a thing. In my opinion they were promised that cameras would have been turned off, or otherwise that video would eventually been burnt because… this was too much.”

Some participants placed emphasis on the context due to the pandemic, both inside and outside the prisons, during the COVID-19 first phases. Particular focus was placed on the failure of reassurance by prison administration to inmates, who could not understand what was happening outside and felt in danger for their health. Among other participants, Serena, a chief educator who has worked in prisons for 32 years, felt negative emotions about this:

“I feel anger because we have not been able to prevent those riots and they happened for a reason: a communication gap. We were already all distressed by a pandemic, but prisoners were not aware of what was going on outside: why we operators couldn’t go visit them, why they couldn’t see their families… and they started rioting over. They felt forced, abandoned and the trust built in maybe years just collapsed.”

Talking about the interpretation of the behavior and the feelings of the policemen of SMCV, Sara, a psychologist with 4 years of experience in prisons of North and Central Italy, stated:

“I think Policemen opted for the “one shot” strategy: ‘I’ll show you who’s in charge here’ […]. It was a tactic to restore order at once. But they were also convinced of ... having a higher power, because there is a bit of this power trip that ... prison is a context where you [the policeman, the operator] think you are on the right side, therefore you can do whatever you want.”

Anna’s argument was in line with the latter: “It was a punishment. They beat everyone, including the inmate in a wheelchair. This means that no matter if you were guilty, a whole category had to be punished”.

After seeing the images of the events, almost all the participants expressed feelings such as shame for being part of the penitentiary system, anger, and sadness. Furthermore, hopelessness and a lack of surprise were expressed, too. Livia, a young psychologist having worked for 5 years in the prison system, stated: “As I saw the images, I felt severe nausea and then I immediately thought: ‘Well, now that I have seen these images, let’s continue to sleep as usual, there is no hope anyway’”. Stella, a social worker who has operated in a Northern prison for 4 years, continued along the same lines:

“It hurt me a lot to see these images, that is… I just looked at them with anger and contempt. And there is this part that leaves a bitter taste in my mouth because I’m angry but not surprised: and this is not good. Like we just know that in prison the inmates take drugs and the cops beat.”

### 3.2. Theme 2: Structural Problems of Penitentiary Police

Participants agreed on viewing more of a structural than a personological problem when talking about police brutality in prisons. Issues arise from the beginning, right from the recruitment and training of new policemen, as underlined by Anna:

“The recruits have courses in effective and persuasive communication… however, those are not experiential while, to say, they do exercises with guns, don’t they? Even for recruitment, psychologists administer the Rorschach or the MMPI, but… candidates know them by heart! They have all the answers already uploaded online. Therefore, you need to change all the selection form, to use new tests.”

The problems continue on trainees’ first hands-on experiences, as Marta, a psychologist with 4 years of experience, expresses:

“New young agents are deployed with a kind of tutor, the senior agent, and… it is obvious that what the senior agent proposes is absorbed by the new one. If someone has the strength to say “no, I’ll think with my head”, better. But the problem is that often a policeman just follows the procedure, the disposition. He executes the orders, without thinking.”

Incapability in terms of negotiation skills can lead to difficulty in dealing with controversies and conflicts, which are the daily experiences of penitentiary police. Roberta, a clinical criminologist who has 11 years of length of service, explained those dynamics as follows:

“If the team of agents works, this will lower the level of conflict, by trying to manage the situation in a dialogical way; if, instead, their modality of managing the conflict situation is “I’m in charge, you have to submit to my orders”, well, they’ll necessarily raise the tone… sometimes it’s not a question of ‘bad apples’, rather of negotiation skills.”

Sara confirmed: “You need a communication channel. Prisoners are as peaceful as they feel cared, not controlled: it’s different. If you talk to them, they feel that they are someone... they already change their mood. Some agents are very good at this”.

However, according to all participants, whether some agents are good is due to personal rather than structural factors. In this sense, Marta denounced an important lack of training for agents: “After the course that they [the agents] do to enter in the force, everything is left to free interpretation. The prison and the administrative system offer nothing in terms of training”.

The lack of training affects various areas. First of all, according to the operators, there is little preparation on the new psychological and cultural dynamics of prisoners, as said by Luciana, a social worker who has worked for 24 years in a prison in Northern Italy:

“You should just walk through a section to understand that there is a negative layer of… Of difficulty… of malaise. Now in prison we have people with severe mental problems… Many foreigners who speak little Italian, so the agents, too, have constantly to deal with very difficult situations.”

The presence of new types of prisoners, as regards origin, ethnicity, or orientation or gender identity, may find closed minds and poor understanding by the agents, as reported by Sara:

“I have followed several inmates with gender identity problems. So, something that is a bit new. Here the comments [from the agents] become cringe. That is... I do not think they are ready to work with, for example, pathologies of this kind; not only are they not prepared, I notice a bit of mental closure on the part of the agents […]. But as well as everything that becomes divergent from the average of the population, so that I am homosexual, that I am African, that I am I feel a woman and… everything that moves a little from the average they find it even more difficult to accept it.”

Talking about the long-standing problems of the prison police, Roberta focused on psychological and group issues never resolved by the police force:

“Agents of the penitentiary police think within a structural dichotomy between good and evil. In the long run, this aspect—which is not trivial—permeates existences. They’re structured in such a way as to categorize, and therefore to put a compartmentalization. This allows you to survive in a situation where good and evil are often blurred, where right and wrong are not it is as clear and categorical as they tell us, where the principle of equity is a strongly nuanced principle.”

Sofia, an educator with a great experience accumulated in 36 years, confirmed this dichotomy: “I know some very good and pacific agent. But they still have this cult of their uniform: ‘I have the uniform: he made a mistake, he’s on the wrong side. I’m better’”.

When episodes of violence by prison officers occur, an important role is played by the trade unions, as stated again by Sofia, talking about SAPPE, one of the most influential penitentiary police unions: “I don’t know if I can tell this but... SAPPE is their cover, and it always has been. The union is perhaps one of the most influential forces in the various games of power that there are in the Prison Administration”.

Another problem is stigmatization among policemen, even when they need help, as stated by Margherita, a psychologist with 19 years of experience:

“It happened to me: ‘So you went to talk to the psychologist? Are you crazy?’. They get tagged right away. It is not easy, even in this sense it is necessary to organize in such a way as to take this dynamic into consideration.”

### 3.3. Theme 3: Structural Problems of the Italian Prison System

Structural problems have been identified in the management of Italian prison facilities that can lead to episodes of violence, as Roberta explained in a good resume of the situation:

“Prison management is overmuch left to the local organization. This creates a sort of self-regulating ‘bubbles’. Which becomes good, if you have a virtuous self-regulation; but it becomes dysfunctional and risky when you have a vicious self-regulation dictated by subjectivity and from the reinforcement that subjectivity receives and legitimizes itself within the group.”

Giulia, a psychologist and educator with more than 35 years of experience in several prisons of Northern Italy, identified two main forces in prisons’ balance of power: “There are two forces: civilian and military. If there is harmony between the prison director and the police commander this will be a great strength for everyone’s health. If the strength of the military is stronger than the civilian, well…”

A theme reported by many participants is the structural slowness of the bureaucracy in prison, and how this can exhaust both prisoners and officers, to the point of fostering episodes of violence. Marta and Roberta offered a glimpse with two proper examples:

“I was listening to an inmate who needed to make a phone call. The number was authorized, but he noticed that one of the 8 digits was wrong—therefore, he would not have really called his house; he pointed this out and the agent replied: “You are authorized to call this number, just call this number”. This is the mentality; it is extremely cumbersome and difficult to change.”

“Think you’re a prisoner waiting for an answer which never arrives. And you spend the days locked in a cell waiting for an answer, which maybe simply got lost because an administrator put your card in someone else’s file. And you wait. At that point, the uniform represents the institution that never gives me answers […]. Therefore, I make myself heard: I scream, I slam, I don’t go back to the cell, I cut myself, I sew up my mouth, I go on a hunger strike… those are all conflictual behaviours asking for the system to take charge of the situation. So, I activate the system but in a dysfunctional way; the only tool I think I have is to mess with it because that’s how I get their attention.”

Bureaucracy is not the only risk factor for escalating violence in prison. Greta, a psychologist who has worked for 30 years in prisons in Central Italy, spoke of Italian prisons as overcrowded “greenhouses”:

“We have young people, in their first detention, very young… and we put them in the cell with the inmate who practically lives in prison. So, as the inmates say, prisons are “greenhouses”, where new offenders are grown. Inmates come out more aggressive than when they entered. And this can also create friction with the Penitentiary police and unleash episodes of violence. When you keep six people with different crimes in terms of gravity in the same cell, it is clear that the weakest will succumb, and this then creates a whole series of tensions even with the agents.”

### 3.4. Theme 4: Proposals of Reforms and Improvements Regarding the Italian Prison System

The solutions proposed by the participants mainly move on two ridges: measures to combat police violence and structural reforms of the prison system. Regarding the first point, Serena said: “A proposal is to put an identification code on the agents’ uniforms, or body-worn cameras… not only because I know who the ‘bad apple’ is. It is also a system that allows me to say “it wasn’t you”, in front of an allegation”.

Prison police officers are seen by many participants as needing help to cope with the various difficulties they experience daily. This, for example, is the thought of Elena, a psychologist who has been working in prison since 2009, regarding the possibility and methods of offering psychological support to prison police officers:

“What I believe should be implemented is support for this profession [penitentiary police]; there should be listening groups, sharing groups, a space in which you bring the experience, the frustration, the sense of helplessness in some cases. And reprocess it. An outdoor space would be extremely functional; precisely because you must reprocess something that happens inside the prison… And also, for avoiding the stigma of the colleagues.”

On a structural level, it is seen as necessary to bring the pedagogical area closer to the security area, making policemen and operators work together, as Marta stated: “Greater sharing between the police command, the pedagogical area and the sanitary area could help both finding the strategies to respond to the needs of the prison population and also to serve as a decompression chamber for all penitentiary operators”. The exchange with the outside is also fundamental, as Giorgia said: “We need to increase all the figures who intertwine the inside with the outside, because seeing people and having productive commitments makes the change. When they commit themselves, they behave and the agents are more serene and able to interact”. Serena, displaying a comparison with the Spanish prison model, proposed a structural reform:

“In some respects, the Spanish model is very advanced. The policemen who work in that system are 15,000, we have 45,000; however, in Spain there are more than 7000 people qualified as operators, that is, educators, psychologists, social workers, mediators… so it does not mean that you empty the prison by removing the policemen, rather you strengthen the prison by letting other figures in. There must be a mass of personnel entering, and replacing the prison police, not in the supervisory tasks, but in the tasks of involvement, of empowerment.”

Roberta continued among the same lines:

“One of the ways is to work with the outside world and then work with opportunity projects. The third sector, voluntary work, that is, giving the possibility of having spaces that give meaning to the time of punishment. A window on the world that also involves agents. In my utopian mentality there is the idea of co-constructed projects where the prison police are part of the re-educational treatment process.”

A final point focused on the architectural structure of Italian prisons and how this affects the process of recovery and reintegration of prisoners, as Alice, a psychologist who has worked in Central Italy’s prisons for over 23 years, said:

“The prison institution should be changed, as was also required by the law, with dynamic surveillance, or open custody, in which it is possible to guarantee the inmate an independent career path and to reduce the physical presence of the police inside the detention areas thanks to cameras and other security measures. However, this is not possible due to the way these [Italian] prisons are built and structured. There are prisons in Norway and Sweden where the architecture allows for dynamic surveillance and there is an effective recovery of prisoners; but we cannot apply the same type of project here in Italy, otherwise it is as if I wanted to put new furniture in a house where there are not even walls. So, what are we talking about? We must start with the modification of the structures.”

## 4. Discussion

The events that took place in the “Francesco Uccella” prison of SMCV triggered a spiral of discussions, both public and private. Combined with the already widespread perception of deprivation and the decay of Italian prisons [1,5], due to the SMCV mass beating, a new awareness of state violence has now risen. With 105 defendants including agents, health workers, and officials of the Department of Penitentiary Administration, this is going to be the single biggest trial against state workers ever held in Italian history [43]. Still, more than 100 policemen will not be indicted as they have not been yet identified [44]. An event of this magnitude led the Italian government to put a structural reform of the prison system among its priorities, mentioning what happened at SMCV as “a defeat of the State” [45].

The aim of this exploratory research was to investigate and to highlight the expert and competent opinions of prison staff regarding incidents of violence in prison. With their stories, participants demonstrated possessing the psychological, relational, and psychosocial skills aimed at responding to a request for help with humanization of care and attention to the other [46]. Their experiences manifested the cognitive skills of critical and creative thinking and problem solving, the relational skills of effective communication and self-awareness, even of their own limits, and emotional skills such as empathy, managing one’s emotions and stress in an extremely complex workplace [47]. Furthermore, with their contributions, the interviewees showed a clear awareness of the state of fragility of the Italian prison population [6]. Their ability to read the context in which they work has brought out a differentiation between the structural difficulties present for decades in Italian prisons, the fragility of the prison population, and the impact that the COVID-19 pandemic has had on both of these factors [6].

The analysis revealed four common themes: the participants’ interpretations and emotions about the events of SMCV; their thought on the structural problems related to the penitentiary police force and the Italian prison system in general; and proposals about reforming and improving the prison system.

Participants’ interpretations about the SMCV events depicted the police actions as a demonstrative act and a way to restore their power, in line with what was already described by Zimbardo and colleagues in their Stanford Prison Experiment [14]. If the goal was to restore the asymmetry of power, the means might have been to eradicate from the inmates any desire for subsequent revolts through a deliberately disproportionate reaction to the riot and, therefore, breaking the bonds between prisoners [48]. The problems which emerged from the results relating to the wall of silence and legitimacy of violence by higher authorities are in line with the international literature which notes how easy it is to systematically cover crimes committed by police forces, favoring a process of secondary victimization [19,49].

Practical measures were mentioned to counter the abuse of physical force by prison police officers. The most relevant was to insert an identification code on the helmets, or cameras on the uniforms (the so-called ‘body-worn cameras’, BWC). In line with what was stated by the participants, international studies [50,51] have shown that, if adequately supported by a ruling class sensitive to their well-being, policemen feel more protected by the BWC; moreover, the same BWCs have favored an improvement in the organizational environment of the police forces that have decided to adopt them.

There is a striking closeness between what was reported in the experience of the participants and what the literature says on the regulatory and cultural aspects of the police forces [20]. These often refer to a narration of the penitentiary police constantly considering itself a frustrated and poorly recognized profession, which is associated with the desire for redemption through a demonstration of strength and power. In the results, the importance of the uniform for agents also emerged. Participants spoke of the uniforms’ symbolic importance in creating a psychological dichotomy fundamental for protection in front of ambiguity [21]. In accordance with the literature [24,25], participants spoke of the structural and staffing difficulties faced by the prison police force and how this leads to psychological problems in agents. In line with the literature [52,53], one of the reasons that emerged most strongly is the lack of training and support for agents, who face their inability to manage critical events with alternative tools to the use of force. As underlined by the participants, the prison population changes concomitantly with historical and social transformations. On the other hand, though, those who deal with security in prisons remain the same policemen as always, with the same skills and awareness as always, by now unable to understand and deal with the numerous changes that have taken place. Dealing with prisoners of different ethnic groups, cultures, or with new and more complex psychopathological problems than in the past, leads policemen to face critical events that either did not exist until recently, or that required less complex management measures. The lack of skills required to confront such events deprive professionals of their sense of adequacy, ability and sense of control, consequently diminishing their psychological or physical well-being [9,10]. Due to the current situation, the cases of psychological problems among police officers are increasing [54]. In line with the literature [55,56,57], the participants see their colleagues of penitentiary police suffering from psychophysical symptoms and yet rarely having access to experts’ help. To this lack is added a distrust, typical of the “police culture” [20], towards the figure of the psychologist: as has already emerged from previous studies (see, for example, [58]), the fear of being stigmatized for the explicit manifestation of suffering, as well as the fear of being judged unable to maintain the service gun, lead agents to refuse help from mental health professionals. In this sense, as emerged from the results, it is crucial to find spaces in which the stigmatization component can be eliminated, bringing psychology to the workplace, rather than requiring the individual agent to enter the psychological setting—as already expressed in the literature on the psychology of law enforcement agencies [23,59].

As far as the structural problems of the Italian prison system are concerned, the participants exposed situations of systemic violence, especially in terms of violence of inertia and bureaucratic violence [18]. Many problems are linked to prison overcrowding and the simultaneous understaffing of social and health workers [1,5]. In Italy, the ratio between prisoners and prison officers has, in fact, been stable for some years at 1.6 compared to a European average of 2.6 [60]; the ratio between prisoners and social-health workers is instead, at the moment, 73 people for each educator, with some institutions reaching a ratio of 200 to 1 [5]. This is one of the reasons for the bureaucratic slowdown much complained about by the participants: the shortages of administrative staff, in fact, have repercussions on the other professional categories, as they force staff who could take care of the inmates “on the field” to devote much of their time to tasks of “office”. Making explicit reference to the Spanish model, some participants underlined the need to break the wall that separates the “inside” from the “outside” in the Italian prisons. Reversing the balance of power between police officers and operators is a desirable reform, too. A more open and community perspective is, in fact, hoped for by most of the participants, in order to dispose of the overcrowding that paralyzes the current system and offer a real path of recovery to prisoners.

The psychologists, educators, and social workers who took part in this study provided extremely valuable thoughts, opinions, and ideas regarding the events of SMCV, the complexity of the Italian prison system, and the problems of the prison police force. Their expertise and competence shed light on the mechanisms of violence and power that occur in closed and total institutions, and at the same time, they were also able to reflect on these problems by offering their personal ideas for change and reform with a view to improving the life of prisoners and operators.

## 5. Conclusions

The reflections that emerged helped to outline the current situation of the Italian prison system more effectively. It is a prison system that is in pain and that is based on too many contradictions still unresolved. The dynamics of power that revolve around and within the prison administration were boldly expressed by the participants, as was their vision on ambiguous facts, on cover-ups. For many, having to explain to an outsider the functioning of their daily work was an opportunity to focus on dynamics they had always seen but perhaps never observed; for others, it was the opportunity to give voice to conflicts, problems, and frustrations. The critical and expert gaze of those psychologists, social workers, and educators who live in prisons every day can offer insights and skills capable of fostering an ever so necessary change of the entire system. It, therefore, becomes necessary to take the words of these women as courageous appeals for a substantial change, which has as its ultimate goal the well-being of prisoners and the well-being of colleagues.

### Strengths, Limitations, and Future Directions

One of the main limitations of the study is the lack of diversification of the sample, both as regards the gender of the participants (100% of the respondents were women), and as regards the profession. The Prison Police Corps was effectively sued for a long time, but no prison police officers were interviewed. Any future research may deal with the same issues but read from the point of view of the prison police.

## Figures and Tables

**Table 1:** Participant demographics.

Pseudonyms	Length of Service	Penitentiary of Origin	Profession
Alice	23	Central Italy	Psychologist
Anna	3	Central Italy	Clinical criminologist
Elena	12	Northern Italy	Psychologist
Ginevra	8	Northern Italy	Clinical criminologist
Giorgia	19	Northern Italy	Educator
Giulia	36	Northern Italy	Educator, Psychologist
Greta	30	Central Italy	Psychologist
Letizia	6	Northern Italy	Social worker
Livia	5	Central Italy	Psychologist
Luciana	24	Northern Italy	Social worker
Margherita	19	Northern Italy	Psychologist
Maria	37	Northern Italy	Social worker
Marta	4	Northern Italy	Psychologist
Roberta	11	Northern Italy	Clinical criminologist
Sara	4	Northern Italy	Psychologist
Serena	32	Northern Italy	Educator
Sofia	36	Central Italy	Educator
Stella	4	Northern Italy	Social worker
